# Surveillance and Reporting of Hospital-Associated Infections—A Document Analysis of Romanian Healthcare Legislation Evolution over 20 Years

**DOI:** 10.3390/healthcare13030229

**Published:** 2025-01-24

**Authors:** Alexandru Coman, Dana Pop, Flaviu Muresan, Florin Oprescu, Shauna Fjaagesund

**Affiliations:** 1 Department of Infectious Diseases and Epidemiology, University of Medicine and Pharmacy “Iuliu Hatieganu”, 400012 Cluj-Napoca, Romania; alexandru.coman@umfcluj.ro (A.C.);; 2 School of Health, University of the Sunshine Coast, Sippy Downs 4556, Australia; 3 General Practice Unit, Faculty of Medicine, University of Queensland, Herston, QLD 4006, Australia

**Keywords:** hospital-acquired infections, Romanian legislation, healthcare policy, infection surveillance, under-reporting, HAI

## Abstract

**Background/Objectives:** The objective of this study is to evaluate the evolution of Romanian legislation related to HAIs. The evolution of healthcare-associated infections (HAIs)’s definitions and surveillance frameworks in Romania reflects progressive advancement in diagnostic criteria, reporting, and prevention. Recent changes emphasize the need for accurate and centralized electronic reporting, inclusion of medico-social and palliative care institutions, and modernized hospital infrastructure standards. However, workforce deficits, insufficient infrastructure, and punitive sanctions remain barriers to effective implementation. **Methods:** This study analyzed publicly available Romanian legislative texts and their evolution, comparing definitions, diagnostic criteria, and surveillance structures. Key informant insights supplemented findings to contextualize legislative impacts. Legislative acts were reviewed sequentially to identify updates in regulatory frameworks and barriers to effective HAI management. **Results:** The legislative evolution demonstrates a shift from basic diagnostic criteria to a comprehensive surveillance framework aligned with European standards. However, challenges persist, including workforce capacity deficits, under-reporting due to fear of sanctions, and infrastructure inadequacies. The prevalence of HAIs remains largely under-reported (4.1%), with studies revealing rates well below the European average (7.1%). Manual and isolated reporting systems further hinder real-time surveillance and accuracy. Legislative advancements in Romania reflect progress in HAI management but highlight systemic barriers that impede effective implementation. **Conclusions:** Collaborative efforts across individual, organizational, and system levels are required to address workforce training, reduce under-reporting, and invest in infrastructure and electronic reporting systems. Promoting a blame-free organizational culture, combined with training, is essential to encourage behavior of accurate reporting and improve HAI prevention strategies.

## 1. Introduction

Hospital-associated infections (HAIs), or nosocomial infections, represent a global public health issue, affecting both developed and developing countries [1,2,3,4,5]. These infections pose major risks to patient safety by contributing to increased post-acute and long-term care morbidity and mortality, and healthcare system financial burden related to infection management [6]. From a regulatory perspective, HAIs are often classified as infections that occur while receiving health care, developed in all health care facility that first appear 48 h or more after hospital admission, or within 30 days after having received health care [5,7]. Globally, HAIs pose challenges to healthcare systems as the most frequent and preventable adverse patient event within hospital-based settings affecting patients and medical personnel [5].

The most common types of HAIs include pneumonia, urinary tract infections, surgical site infections, and bloodstream infections [1,2,8,9]. Globally, the risk factors associated with HAIs include recent antibiotic use, use of invasive devices treatment in intensive care unit, pre-operative illness severity, and a history of respiratory tract infections [10,11]. Certain pathogens, such as *Klebsiella pneumoniae*, *Acinetobacter baumannii*, and *Pseudomonas aeruginosa*, are commonly associated with HAIs and are more likely to exhibit antimicrobial resistance [10,12,13].

In Europe, it has been reported that HAIs cause 16 million additional hospitalization days each year, with costs exceeding 7 billion euros annually [4]. The European Center for Disease Prevention and Control estimated that 4.3 million hospitalized patients in the European Union (EU) and European Economic Area acquire at least one HAI every year, with one in three microorganisms confirmed as bacteria resistant to more than one (multiple) antibiotics, thus limiting treatment options and increasing mortality and morbidity risk for infected patients [14].

In Romania, a middle-income country of the EU, HAIs and related multidrug-resistant infections are recognized as a serious public health issue [15]. Despite such concerns, healthcare system data on the epidemiology of such infections in Romania are limited [15] and under-reported compared to other EU countries [16]. The reported prevalence rates of HAIs in Romania are estimated to only represent 0.2–0.25% of actual cases, with *Clostridioides difficile* as the frequently detected pathogen responsible for HAIs [4]. Romania faces several challenges, including under-reporting of HAIs, outdated hospital infrastructure, and a high prevalence of multidrug-resistant bacteria. Due to under-reporting, the official prevalence rate is lower compared to the EU average of 7.1% [14,15].

Rapid, sensitive, and specific methods are needed for the identification of bacteria causing HAIs. Traditional culture methods have limitations, and there is a growing interest in developing rapid detection methods [15]. Studies conducted in Romania emphasize the need for improved infection prevention and control strategies, better reporting, and an updated hospital infrastructure to reduce the incidence of HAIs through better understanding and control [4].

Given the aforementioned, an in-depth analysis of legislative frameworks governing infection prevention, surveillance, and control in Romania is warranted as a starting point in understanding the regulatory processes, which impact the healthcare system and hospital practices and support mechanisms to prevent HAIs. Such an analysis can identify areas of regulatory improvement to increase patient safety to inform future practices to reduce HAIs in Romania and potentially other similar middle-income EU country hospital-based settings.

The objective of this study is to evaluate the evolution of Romanian legislation related to HAIs, focusing on regulatory development over time and impact on the healthcare system, hospital and medical personnel practices, using publicly available documents from primary data sources.

The main research question addressed in this paper was “How has Romanian legislation related to healthcare-associated infections (HAIs) evolved over time, and what impact has this evolution had on the healthcare system, hospital practices, and medical personnel?”

To address this overarching question, this study proposes the following sub-aims:

- Identify and document key legislative acts, policies, and regulations pertaining to HAIs in Romania from their inception to the present.
- Analyze the context and motivations behind the introduction or amendment of these legislative measures.
- Evaluate how effectively these HAI-related legislations have been implemented within Romanian healthcare institutions.
- Examine changes in hospital infection control practices and protocols resulting from legislative developments.
- Identify any gaps or shortcomings in the current legislative framework concerning HAIs.

## 2. Materials and Methods

The research consisted of three stages: legislation collection, data extraction, and legislation review.

Firstly, we collected the necessary information by accessing and downloading all relevant publicly available legislative texts from iLegis.ro and identifying relevant sections from each normative act, including Order No. 994 of 10 August 2004, Order No. 916 of 27 July 2006, Order No. 1.101 of 30 September 2016, and Law No. 3 of January 2021.

Secondly, we textually extracted and analyzed the definitions and diagnostic criteria for HAIs from each normative act for comparison to identify legislative developments and updates over time.

In the final stage, we focused on extracting and analyzing the organizational and procedural structure, comparing the surveillance and reporting of HAIs to evaluate the responsibilities of institutions and personnel, including the impact of normative acts over time, with a focus on the surveillance (prevalence and reporting) of HAIs before and after practice implementation, identifying barriers and enablers to effective HAI management and its impact on national statistics.

To ensure alignment in definitions and diagnostic criteria for HAIs across different normative acts, authors evaluated the continuity and additional clarifications sequentially by the legislative in-force date of each normative act. Two researchers, AC and FO, completed the qualitative analysis and differences in opinion were discussed until a consensus was reached. If consensus could not be reached, a third researcher, SF, was available to ensure a majority opinion was reached. Key informants were utilized as necessary to provide broader context to review findings.

## 3. Results

The results are presented in the following structure: legal definitions of relevant terms and policy improvement over time and impact of policy on practice.

The definition and surveillance of HAIs in Romania have evolved over time, reflecting changes in medical practices and surveillance requirements. Analyzing Order No. 994 of 10 August 2004, Order No. 916 of 27 July 2006, Order No. 1.101 of 30 September 2016, and Law 3 of January 2021, the following evolution was observed and documented.

**Order No. 994 of 10 August 2004 (Ministry of Health, 2004).** This order defined HAIs in the context of nosocomial infections and focused on clinical and microbiological criteria for diagnosis. The definition was oriented towards specificity of diagnosis where emphasis was placed on detailed clinical criteria and microbiological confirmations, such as positive microbial culture from relevant samples or the presence of characteristic clinical signs without other evident causes. Furthermore, the legislative document details different types of nosocomial infections, including urinary tract infections, nosocomial pneumonia, surgical wound infections, and others, each with specific diagnostic criteria.

Importantly, for an infection to be proven as nosocomial, current legislation requires there must be no evidence that the infection was present or incubating within patients at the time of hospital admission. In comparison to current practice, this criterion is often difficult to verify, as the incubation period of infectious diseases is rarely fixed, and delays in diagnostic testing can affect the ability for hospitals to effectively diagnose pre-existing infections on patient admission. Additionally, in practice, diagnostic procedures can be hampered due to a lack of medical personnel to support surveillance, which is further compounded by insufficient training in identification and knowledge of infectious diseases and the necessary laboratory infrastructure to support immediate diagnosis. Inpatient infections acquired are often diagnosed after discharge (e.g., newborn infections resulting from passage through the genital tract and diagnosed after discharge). The event of hospital-based births is a common example of how post-diagnosis of mothers or children may or may not be classified as a nosocomial infection. Illnesses associated with a complication or extension of an infection present at admission are not considered nosocomial if the pathogen has not changed or if the signs do not indicate a new infection, as well as transplacental infections in newborns (e.g., herpes simplex, rubella, syphilis, cytomegalovirus, toxoplasmosis) that become evident shortly after birth. Each case of nosocomial infection must be proven to be due to hospitalization or outpatient medical care in healthcare units.

Classifying infections as nosocomial is challenging in this context, as the causal relationship is often difficult to prove (e.g., risk factors cannot always validate, nosocomial infections may have multiple causes and exposures, and laboratory capacity for microbiology is insufficiently developed to allow genotyping of microbial isolates and establishment of filiation). In the absence of available conclusive evidence, the tendency of hospitals and medical personnel responsible for surveillance and reporting of nosocomial infections is to find plausible alternative explanations, to classify the infection as a complication or extension of an infection present at patient admission, rather than a nosocomial infection. Under-reporting is evident within available national statistics (incidence rate below 1%, most hospitals did not report or reported an incidence of 0%).

Key informants agreed and offered explanation of under-reporting of nosocomial infections was due to several reasons. Firstly, both hospital management and public health authorities often discourage reporting, as increased nosocomial infection reports trigger regulatory controls, which often use punitive sanctions against medical personnel, including doctors and nurses who are needed for future delivery of care. Secondly, as part of these imposed controls, the primary activity of the hospital epidemiologist is to collect sanitation samples for clinical microbiology testing. These sanitation tests are time-consuming and create additional financial burden on hospital and public health resources. Thirdly, medical personnel, often at individual risk of sanction, are further discouraged to investigate and report nosocomial infections when the information obtained from testing does not have any scientific or practical delivery-of-care importance on decisions regarding patient isolation and decontamination (e.g., cleaning and disinfection) of the hospital environment to prevent further harm and improve post-operative health outcomes.

**Order No. 916 of 27 July 2006 (Ministry of Public Health, 2006).** This order maintains continuity with previous definitions but brings additional clarifications and surveillance structures. Details were added regarding the diagnosis of certain types of infections, such as surgical wound infections and nosocomial pneumonia, specifying the necessary conditions for confirming diagnoses. The legislation introduces a more structured framework for surveillance and reporting of HAIs, emphasizing the importance of accurate recording and reporting of infection cases.

In the 2006 legislation, a nosocomial infection is defined as an infection contracted in healthcare units with beds (public and private) and refers to any infectious disease that can be clinically and/or microbiologically recognized for which there is epidemiological evidence of contraction during hospitalization and/or associated medical act or medical maneuvers, which affects the patient (due to the medical care received) or the healthcare personnel (due to their activity), and is further linked by incubation to the period of medical care in the identified unit, regardless of whether disease symptoms appear during hospitalization.

The legislation maintains the “nosocomial” aspect of infections, namely the requirement for epidemiological evidence of infection contraction during medical maneuvers or acts. Additionally, the definition includes infections that occurred in medical personnel. Key informants explained regulatory confusion with what defines reportable occupational exposure (what part or activity of the job is included), providing contextual examples of common work-related accidents (e.g., needlestick injuries) and delayed disease onset (e.g., conditions arising over time due to one or more job-related exposures).

The epidemiological triad, which is comprised of the source, transmission route, and receptive person or population, is often difficult to establish in practice. Key informants further contextualized microbiological screening at patient admission as frequently absent, noting the contributing factor of varied incubation periods for different infectious diseases, which overall makes it difficult to establish the source of nosocomial infections in patients or medical personnel who may subsequently contract disease. Many hospitals do not have quarantine capacities to effectively isolate colonized or infected patients. Formal isolation procedures do exist, yet in practice, medical personnel are often insufficiently trained in standard adherence and lack understanding of additional necessary precautions to address nosocomial transmission routes. Additionally, this update in legislation did not focus on the immune status of the receptive population, another key factor of the epidemiological triad, in which key informants describe as rarely known, despite a significant proportion of admitted patients with immunocompromised status, belonging to extreme age groups (e.g., newborns or elderly population), and with a history of multiple hospitalizations.

**Order No. 1.101 of 30 September 2016 (Ministry of Health, 2016).** This 2016 legislation reflects a modernized and integrated approach to managing HAIs as follows. Emphasis is placed on integrating infection surveillance into the broader framework of quality management in hospitals, highlighting the importance of electronic reporting and continuous monitoring. The document details specific methodologies for surveillance and prevention of HAIs, emphasizing the need for a single electronic registry for monitoring infections in each healthcare unit. The responsibilities of different levels of management in preventing and controlling HAIs are clearly defined, including the role of public health directorates and infection prevention services.

The legislative framework established by OMS 1101/2016 is significantly modernized compared to previous legislation. The concept of “nosocomial infection” is replaced by “healthcare-associated infection,” with the definition now linked to European legislation (Decision No 2119/98/EC of the European Parliament and of the Council), creating the premises for achieving interoperability at the EU level and resolving some diagnostic and reporting issues.

For the purpose of surveillance, namely reducing the incidence of healthcare-associated infections and their costs, the legislation establishes clear objectives for the first time, such as increasing the interest of medical personnel in detecting, reporting, and ultimately reducing infections; documenting the prevalence and describing the distribution of infections by departments and types of infections, as well as monitoring trends; and identifying departments that require intensive and sustained prevention programs and evaluating the impact of implementing preventive programs.

It was thus legislated that in healthcare units with beds (e.g., hospitals—healthcare units with beds), surveillance systems with specific requirements are established with the implemented elements of simplicity (easy to implement for staff), affordability (low costs for the healthcare unit),flexibility (easy to adapt as the situation requires), acceptability (active participation of medical personnel), collection (of relevant, quality data), standardization (by using case definitions from European legislation combined with a unique national methodology aligned with international practices), and sensitivity and specificity (increasing the number of correctly reported infections by using case definitions). Additionally, this legislation prompted the introduction of related and measurable performance indicators to facilitate compliance.

**Law No. 3 of 8 January 2021 (Romanian Parliament, 2021).** This act provides a consolidated legislative framework for the prevention, diagnosis, and treatment of HAIs. According to this legislative document, HAIs are defined as infections acquired during the provision of medical services that were not present (and not in the incubation period) at the time of the patient’s admission. The law details the obligations of healthcare units and medical personnel, emphasizes the need for a national surveillance system for HAIs. It also specifies sanctions for non-compliance with prevention and control measures. Additionally, the law introduces new standards for healthcare infrastructure, such as ensuring isolation rooms for patients with antibiotic-resistant infections.

Law 3/2021 completes the provisions of OMS 1101/2016, brings clarifications, and represents a significant extension of interest in diagnosing, reporting, and preventing HAIs. The legislative act defines HAIs as infections that occur in patients receiving care in healthcare units, within hospital and/or outpatient medical care, medico-social institutions, and palliative care institutions, or who have recently received such care and were not manifest or in incubation at the time of admission. The modification is significant; in addition to stipulating the obligation to monitor, prevent, and report HAIs in healthcare units with beds (hospitals), the legislative also further defines this type of activity to include healthcare units as any institution providing medical care in continuous hospitalization, day hospitalization, or outpatient care, including medico-social and palliative care institutions (residential centers for the elderly, chronically ill in the terminal phase, residential centers for the elderly, for minors, and for people with disabilities).

Provisions related to this legislation are also contained in a technical document that regulates the design and construction standards of hospitals, particularly in the context of financing the modernization of existing hospitals and construction of new units. These standards aim to prevent, diagnose, and treat HAIs by integrating measures into the design and organization of hospital infrastructure to minimize the risk of contamination and pathogen transmission.

One key standard is the segregation and organization of medical spaces based on the strict compartmentalization based on contamination risk, such as septic, aseptic, and sterile zones. Internal circulation paths are to be planned, so the routes of patients, medical personnel, and materials are clearly defined and segregated to prevent pathogen contamination and transmission risk. Another important measure is the use of physical and hygiene barriers, including filters between areas with different contamination potentials. Investment in specialized equipment is outlined as necessary for the treatment, cleaning, disinfection, and sterilization of all hospital components, including air, water, effluents, and medical instruments.

The control and monitoring of the hospital environment is also addressed with adequate ventilation, heating, and air conditioning systems designed to limit the concentration of airborne pathogens (e.g., hygiene filters). Water systems are regulated to maintain consistent temperatures, which prevent bacterial proliferation (e.g., hot water above 51C and cold water below 20C), alongside adequate chlorination measures, to prevent pathogen exposures, such as legionella. Furthermore, medical waste, including infectious, sharp, anatomical–pathological, chemical, and radioactive materials, is to be managed through segregated collection and treatment in accordance with specific regulations to prevent contamination and ensure safe disposal.

## 4. Discussion

The recent evolution of HAIs’ definition in the regulatory standards reflects significant progress in clarifying and detailing diagnostic criteria for identification, classification, and reporting of nosocomial infections, as well as in the physical and organizational structure for surveillance and prevention [4,14,15,16]. This includes expanded measures of electronic reporting [14] and delegation of responsibilities of HAI management to any healthcare unit provisioning medical care [16], including hospitals and medico-social and palliative care institutions [4,14,16]. These legislative amendments strengthened Romania’s standards aimed to prevent and reduce nosocomial infections from a regulatory viewpoint [15,16]. However, workforce capacity deficits, infrastructure limits, inconsistent data handling, and punitive surveillance sanctions [4,14,15,16] are all identified concerns within individual-, organizational-, and system-level HAI management that may impact the effective implementation of recent changes to Romanian legislation into practice (See Table 1 for details of changes).

The prevention, reduction, and reporting of HAIs involves available workforce capacity within healthcare units to implement and maintain standards. This includes having adequately trained on-site medical personnel (specialist doctors in epidemiology and nurses in infection control) to effectively integrate changing HAI standards, including reporting, into clinical practice [1,3,7,16,17]. Furthermore, overburdening trained medical personnel with administrative and clinical tasks can also make it challenging for them to devote proper attention to reporting nosocomial infections [1,4,15,18].

Organizational culture and fear of sanctions against medical personnel may also play a significant role in the under-reporting of HAIs, resulting in data suppression and resistance [4,14,15,16,17]. As a result, medical personnel may become reluctant to report HAIs to regulators, even if they are aware of their occurrence and the severity of the outcomes, particularly if they feel if such action will not invoke systemic change to reduce patient harm [4,14,15,16,19]. Additionally, medical personnel challenged by systemic ethical dilemmas may experience higher rates of occupational stress contributing to increased burnout [16,19] and disengagement [14,19] and/or psychological injury [4,16,19].

The lack of investment in the diagnostic infrastructure is another significant barrier in the implementation of HAI legislation in Romania. Frequently, hospitals in the country face shortage of equipment and consumables necessary for the diagnosis and reporting HAIs [20]. Screenings may not be performed to all patients due to limited resources and infrastructure in some medical units. Inadequate infrastructures that fail to meet modern standards, including buildings that were not purpose built as hospitals, may lead to difficulties in monitoring and under-reporting of infectious viral infections [1,15,19,21]. For example, hospitals with insufficient laboratory facilities may be unable to [1,4,19] or need more time to perform the tests to diagnose HAIs [4,15], leading to higher rates of post-operative patient mortality, prolonged hospital stays [15,19], and under-reporting (absent, inaccurate, and incomplete testing) leading to poor understanding of prevalence and trends to effectively prevent and control infections [4,15,19]. Additional research identified the significant impact of these infections on healthcare settings in other parts of the world and call for improved infection control measures and the development of new treatment strategies to combat these resistant infections [22]. Additionally, lack of standardized protocols would also lead to under-reporting.

From an ethical and practical perspective, under-reporting of HAIs remains of current and future primary concern [4,15,16,23]. Despite legislation, the under-reporting of HAIs in Romania is still an issue. The results of a prevalence study conducted in Romania in 2023 [24] documented this aspect. Out of the total patients included in the study, 674 patients had active HAIs, resulting in a prevalence of 3.1% (95% CI 2.5-3.8%). The corrected prevalence after validation was 4.1% (95% CI 3.3-5.1%), well below the European average of 7.1% [13]. The highest prevalence of HAIs was recorded in intensive care units (ICUs) with 14.9%, followed by medical wards (3.4%) and surgical wards (2.5%) [4]. The most common HAIs were gastrointestinal infections, for which there is a distinct methodology for surveillance and reporting (28%), respiratory infections (24%), and urinary tract infections (16%) [16]. Notably, septicemia, peripheral and central catheter infections, and surgical wound infections, which European hospitals report, are missing from the top list in Romania, suggesting these types of infections are not being routinely identified, tested, and/or reported [16].

### Recommendations for Practice

These legislative changes have significant implications for the Ministry of Health’s personnel policy. It is recommended a gap analysis audit of the existing human resource capacity within current and future clinical environments is needed [1,4,5,19]. Specifically, identifying gaps in knowledge and leadership capacity of medical personnel and organizational management to support effective supervision and prevention HAIs [1,3], including adaptability to adjust procedures changing standards to potential risks [25]. Identifying current and future workforce capacity needs can also inform targeted investment in training programs, for all identified categories of workers, to ensure a qualified individuals in healthcare units can understand and implement standards within their scope of practice or responsibility [3,19,25]. Partnerships involving universities and other educators specializing in evidence-based infection prevention, control, and surveillance can build medical personnel capacity to progress the implementation of effective HAI management [5,14,19,25] more effectively than sanction enforcement alone.

Based on our findings from key informants and the relevant supporting literature [5,18], we suggest systemic improvements, focused on education, and behavioral changes as key strategies to improve HAI management over sanction-based approaches, which negatively impact medical personnel and organizational culture within healthcare units. Reliance on strict penalties for compliance can decrease surveillance engagement by medical personnel [14,19] and may lead to unintended workforce capacity and productivity issues caused by psychological distress or burnout from data suppression and reporting resistance [16,19].

Accurate reporting of HAIs is a key component to the systemic prevention and mitigation strategies. Not all Romanian hospitals use the same database format, which requires data to be sent monthly to local county Public Health Directorate for collation with other reporting healthcare units, before transmittal to Regional Public Health Centers for final regional amalgamation and centralization at the National Institute of Public Health [16]. The current system is administratively burdensome and time-consuming and affects the real-time data entry processes, which may also contribute to under-reporting. Research confirms that manual or paper-based reporting systems are more vulnerable to errors, delays, and/or omissions than electronic approaches [14,15,17]. It is recommended that the Ministry of Health reviews these systemic barriers and considers investment in a harmonized electronic reporting system and integrated automation of notifiable adverse events [26] for all regulated healthcare units to increase record accuracy and enable faster HAI prevention and control response. Additional suggestions are included in Table 2.

As a limitation, it must be noted that this study included only Romanian legislation. There was no comparison with HAI legislation from other countries, and findings and recommendations are only applicable for Romania. However, this study has analyzed all the relevant legislation for the last 20 years and can be used as a model by policy researchers from other countries.

## 5. Conclusions

The analysis indicates that current Romanian HAI legislation is fully aligned with the European standards. The legislative analysis shows a clear progression in addressing HAIs, evolving from basic definitions and clinical criteria to an integrated and modern framework for surveillance and reporting. The latest legislation brought significant modernization by introducing electronic reporting and continuous monitoring, in line with European standards. However, the success of implementing the legislation depends on several key interventions, such as planning resources, adequate training of medical personnel, and ensuring an organizational culture of a safe and blame-free environment that encourages accurate and complete reporting of HAIs requiring individual-, organizational-, and system-level interventions and collaborations.

## Figures and Tables

**Table 1:** Summary of legislative changes, challenges in practice and identified level factors.

Legislation	Key Changes	Challenges in Practice	Level Factors
**Order No. 994 (2004)**	Defined nosocomial infections using clinical and microbiological criteria Focus on specific diagnostic criteria for different HAI types	Difficult to verify if infections were present or incubating at time of admission due to variances in pathogen incubation periods Insufficient medical personnel and training to support surveillance Limited laboratory infrastructure to confirm nosocomial infections High levels of under-reporting evident	**Individual**: Lack of training; fear of sanctions discouraging reporting **Organizational**: Limited staffing and laboratory infrastructure **System**: Ambiguous criteria for certain nosocomial infections; punitive regulatory controls
**Order No. 916 (2006)**	Expanded on previous definitions; added more structured surveillance and reporting requirements Included infections in healthcare personnel from occupational exposure Emphasized epidemiological evidence for nosocomial infections	Epidemiological triad (source, transmission route, and receptive population) remains difficult to establish Lack of microbiological screening at admission Limited quarantine and isolation capacities Insufficient training on transmission routes and precautions Immunocompromised patients, and other patient factors increasing HAI risk, often not identified	**Individual**: Insufficient training in infection control; limited understanding of precautions **Organizational**: Inadequate quarantine and isolation facilities **System**: Complexity of epidemiological triad and unclear guidelines for occupational exposure (e.g., needlestick injuries and delayed effects)
**Order No. 1101 (2016)**	Modernized approach linking surveillance to healthcare unit (e.g., hospital) quality management Aligned legislation to closer in alignment with EU requirements Emphasized electronic reporting and monitoring using a centralized, single national registry Introduced measurable performance indicators for compliance and surveillance Standardized case definitions and methodologies for consistency Focused on prevention programs and evaluating their impact	Implementation challenges due to affordability and acceptability issues among medical personnel Resistance to adopting electronic systems Difficulty in achieving full standardization and sensitivity in reporting	**Individual**: Resistance to change; lack of motivation to adopt electronic systems **Organizational**: Limited funding for prevention programs and electronic systems **System**: Inconsistent implementation of standardized methodologies; lack of alignment with performance indicators
**Law No. 3 (2021)**	Consolidated framework for HAI prevention, diagnosis, and treatment Expanded scope to all healthcare units, adding outpatient, medico-social, and palliative care institutions Introduced new hospital design standards to prevent pathogen transmission (e.g., segregation into septic/aseptic zones, ventilation systems, and hygiene barriers) Established national obligations for surveillance and stricter penalties for non-compliance	Implementation of infrastructure standards may require significant funding and time Segregated air systems for isolation areas not explicitly mandated Monitoring compliance in smaller institutions (e.g., outpatient or residential centers) remains challenging due to workforce capacity Balancing sanctions with incentives to encourage reporting	**Individual**: Challenges in adhering to new standards without adequate training **Organizational**: High costs for infrastructure upgrades **System**: Limited resources for monitoring compliance in smaller or non-hospital settings

EU: European Union; HAI: Hospital-associated infections.

**Table 2:** Proposed areas of focus for future policy revisions and initiatives.

Area of Focus	Study Conclusions	Future Recommendations
Legal definitions of relevant terms	Older legislation laid the groundwork for diagnostic criteria and introduced the first surveillance structures. In the present, the legal definitions of relevant terms in the Romanian legislation are aligned well with the European standards.	Continue monitoring the European and other internation standards in order to consider potential revisions in the future to stay align with the international standards from a legal point of view.
Future policy legislation improvement	Hospital managers and medical personnel are currently directly responsible for HAI surveillance and reporting.	Further changes could be made to financial instruments specified by legislation to encourage investments in staff capacity building and the medical infrastructure.
Impact on practice	In the last 5 years, the legislation prompted government investments for improve isolation and control in hospitals.	The next funding initiatives aligned with legislation should encourage investments in the digital infrastructure for surveillance and reporting.

## Data Availability

All legislation analyzed is publicly available.
